# The effect of recess number and conical shape on hydrostatic bearing power losses

**DOI:** 10.1038/s41598-025-26402-5

**Published:** 2025-11-22

**Authors:** Marwa M. El-Sayed, Masoud Ibrahim, Ahmed. S. A. Abou Taleb

**Affiliations:** 1 Department of Mechanical Engineering, Faculty of Engineering, Fayoum University, Fayoum, 63514 Egypt; 2 Faculty of Engineering, Al Ryada University, El Sadat City, 32897 Egypt

**Keywords:** ANSYS CFD, Hydrostatic bearing, Pocket shape, Power losses, Conical shapes, Energy science and technology, Engineering

## Abstract

Axial loads on machinery are commonly carried by hydrostatic thrust bearings. To ensure sustained performance, designers need to balance the requirement for efficient pumping power with the high load-carrying capacity. This requirement is a common challenge. For the purpose of studying the pumping power losses of hydrostatic thrust bearings, this research presents a numerical analysis of the recess shape design effect. The ANSYS workbench software has been used to implement the numerical simulation. Rectangular and circular pockets with two and four recesses are analyzed for power losses and film thicknesses using FVM and the Navier–Stokes equations. Study results indicated that the number and shape of recesses had a significant effect on power losses.

## Introduction

The majority of industrial applications have utilized thrust bearings with externally pressurized systems because of their desirable design characteristics. Such kinds of systems are valid owing to hydrostatic thrust bearings often need lubricated by external sources. Over 40% of the energy used by machine tools is consumed by the fluid system. To minimize this consumption, overall pad hydrostatic thrust bearing characteristics must be enhanced from the recess’s perspective^1^. Power loss elimination is still essential for bearing improvement in today’s technological progress. Michalec et al^2^ studied a novel two-parameter method by using CFD to predict the ideal hydrostatic bearing parameters, pad position, and area of recess, and calculating the factors of bearing performance with the MATLAB program. Results indicated that the suggested model decreased the loss by 20% when compared to the traditional method. Singh et al^3^ used an electro-rheological (ER) lubricant to numerically examine hybrid thrust bearings, aiming to maximize the textured surface and minimize frictional power loss. According to the results, the use of dimples significantly decreased (−48.8%) and improved (+ 4.3%) the frictional power loss in textured bearings. Wilkes et al^4^ Investigated the effects of spiral-groove thrust bearings and bump-foil ambient pressure. Results demonstrated that the power loss was significantly greater and that the spiral-bearing capacity is insensitive to pressure. To study the impact of different shapes (circular, spherical, conical, and square), Agrawal et al^5^. introduced a hybrid spherical thrust bearing model. Results showed a 24% decrease in frictional power loss. A two-lobe bearing type that is made to minimize frictional power loss was studied by Hagemann et al^6^. This design enhances the test bearing’s frictional power loss by 37.2% by replacing the bearing’s sliding surface portion with open spaces to create very little friction in its boundary layers. Canbulut et al^7^ examined how the lubrication of a hydrostatic bearing is affected by surface roughness. The results demonstrated that surface roughness had an impact on frictional power and that loss dropped as the supply increased. Theoretically, Wang et al^8^ analyzed the stiffness, load-carrying capacity, and power consumption of hydrostatic thrust bearings. The water hydrostatic thrust bearing properties have been reported to be influenced by the characteristics of the fluid and elastic materials. Hong et al^9^ studied how power losses under mixed conditions of friction were affected by the bearing balance of piston and recess ratios. The findings confirmed the belief that enhancing the resistance of external tilting moments might reduce power losses. An artificial neural network (ANN) model was presented by Canbulut et al^10^ to solve the challenge of hydrostatic bearing frictional power loss in pump equipment. The outcomes showed the beneficial effects of the proposed model in analyzing these systems. Ferguson et al^11^ carried out an experimental investigation into the influence of thrust bearing oil viscosity on power loss. The bearing power loss reduced as the oil viscosity dropped, based on the results. To minimize the overall power losses of hydrostatic journal bearings, SALEM et al^12^ studied the variables of optimal design. The findings showed that load carrying capacity, circumferential and axial land width, and the area to pressure ratio are the variables that are concerned. A computer strategy for optimizing the multi-recess of hydrostatic journal bearings has been provided by CUSANO et al^13^. The design data from the results was applied to select the restrictor’s size and recess geometry to reduce the overall power loss. Veauce et al^14^ performed an experimental study on the power losses of rolling element bearings, concentrating on the consequences of oil bath lubrication. The results showed that the oil level rose as a result of the bearing’s increased drag, which in turn triggered an increase in power losses. Wasilczuk et al^15^ constructed a variety of tilting pad thrust bearing systems with direct supply, which enabled more appropriate lubricant introduction into the oil gap and a decrease in power loss. The groove at the pad leading edge could improve the bearing properties without increasing flow, according to the results. By taking into account characteristic bearing parameters such as bearing pressure distribution, load carrying capacity, power losses, friction torque, and temperature, Bedairia et al^16^ proposed a circular and rectangular hydrostatic bearing recess shape. According to the results, the circular recess pad was implemented the best in all aspects. Kumar et al^17^ established the ideal micro-groove parameters of the hybrid bearing configuration and investigated the impact of surface texture on the hybrid thrust performance system. Owing to the results, using a full-section micro-groove can increase bearing efficiency by minimizing power losses and improving fluid reaction. Untaroiu et al^18^ solved the multi-variable genetic algorithm and selected the groove’s depth. To mitigate power loss and enhance capacity, the groove geometry was optimized. Yang et al^19^ examined the hydrostatic oil pad and measured the impact of oil viscosity, flow rate, and friction on those factors. Doshi et al^20^. used MATLAB to create a mathematical model for a circular hydrostatic bearing, aiming to build a bearing with large capacities and low hydrostatic friction. The findings indicated that as film thickness increased, so did power loss. In the current study, the effect of hydrostatic bearing reses geometry (number and shape) on power losses will be investigated. Both conventional and conical shapes of rectangular and circular pads with two and four recesses were analyzed to evaluate the impact of shape and the number of recesses on power losses. Variation of oil film thickness is another factor in the current study. A quantitative study was conducted on the effect of double and quadruple cavity suggested shapes on reducing losses in hydrostatic thrust bearings. In addition, a simulation work is carried out to show the pressure distribution of all proposed designs and analyze their impact on power losses using the FVM technique.

## Methodology

**Fig. 1:**
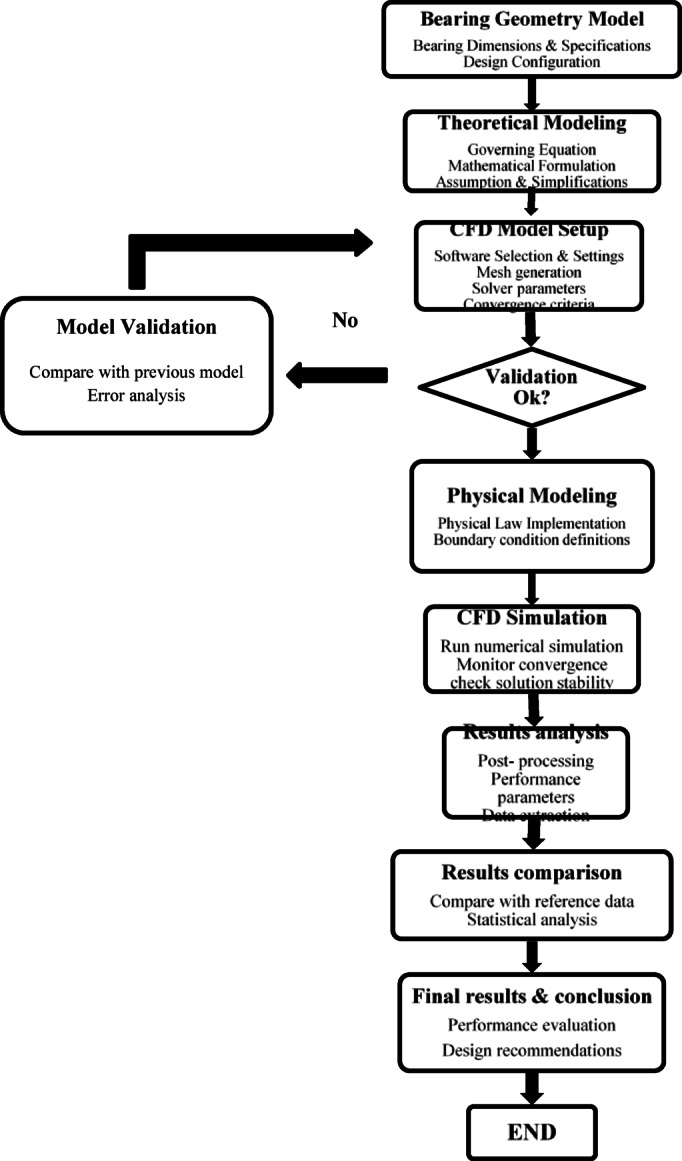
Research methodology flowchat.

Hydrostatic thrust bearing performance is studied in this paper using a systematic computational approach. As seen in the process flow in Fig. 1, the flowchart shows the CFD simulation techniques with theoretical modeling. Bearing geometry configuration, theoretical and physical modeling, CFD setup, model validation, simulation execution, and results analysis are the seven stages of the process.

### Hydrostatic thrust bearing geometry model definition

**Fig. 2:**
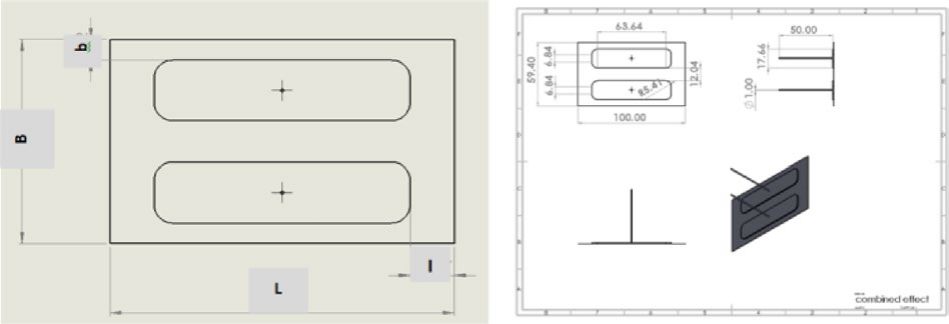
Rectangular hydrostatic bearing pad.

The shape of a rectangular pad used in the HTB is illustrated in Fig. 2. This configuration is 100 mm long and 59.4 mm wide. The restrictor, which feeds water into the hydrostatic recess pad, was chosen to have a diameter of Dc = 3 mm and a length of 50Dc. The pad area, which is about 5940 mm^2^, is selected as a reference for calculating the bearing geometric characteristics. The nomenclature l refers to the length of the bearing sill, and b refers to the sill width.

#### Recess shapes

To study the effect of fluid film thickness and recess shape on loading capacity and power losses, simulations of hydrostatic pads with various recess shapes (Table 1) were conducted. All dimensions in Table 1 are physical values (in meters) of all designs.

**Table 1:** Recess configurations and dimensions of pads (m).

Design	Dimensions, m	Shape	Design	Dimensions, m	Shape	Design	Dimensions, m	Shape
Design I	l = 0.00803, b = 0.01277		Design II	l = 0.00602, b = 0.01252		Design 5	l = 0.00924, b = 0.00854	
Combined effect	l = 0.00803, b = 0.01277		Design III	l = 0.00602, b = 0.01252		Design 5 with cone	l = 0.00924, b = 0.00854	
Design 4	l = 0.000765, b = 0.021065		Design 6	l = 0.000525, b = 0.020535		Design 6 with cone	l = 0.000525, b = 0.020535	
Design 4 with cone	l = 0.000765, b = 0.021065							

*Numerical modeling and Governing equations* The Navier–Stokes (N-S) equations control the fluid flow inside the hydrostatic bearing. Under certain conditions, the N-S equations for thin-film lubrication issues can frequently be reduced to the well-known Reynolds equation [citation]. Nevertheless, the Reynolds equation assumes a one-dimensional pressure gradient throughout the film thickness and ignores fluid inertia, which can result in errors, especially close to recess margins. To avoid the drawbacks of the Reynolds approximation and provide a more thorough and precise prediction of the bearing’s performance, this study uses a Computational Fluid Dynamics (CFD) approach that directly solves the entire (N-S) equations. Both theoretical (Reynolds) and computational (CFD) methods are based on the same Navier–Stokes foundation. However, they have distinct analytical goals: one is to provide a theoretical understanding, while the other is to provide a thorough, realistic simulation. It is produced using the following NAVIER- stock equations: For a compressible flow, applying conservation of mass is [21]:

3 $$\because \frac{\partial \rho }{\partial t}=\frac{-\partial (\rho u)}{\partial x}-\frac{\partial (\rho v)}{\partial y}-\frac{\partial (\rho w)}{\partial z}$$

4 $$\therefore \frac{\partial }{\partial x}\left(\frac{{\rho h}^{3}}{12\mu }\frac{\partial p}{\partial x}\right)+\frac{\partial }{\partial y}\left(\frac{{\rho h}^{3}}{12\mu }\frac{\partial p}{\partial y}\right)=\frac{1}{2}\frac{\partial }{\partial x}\left(\left({U}_{a}+{U}_{b}\right)\rho h\right)-\left(\rho {U}_{b}\frac{\partial h}{\partial x}\right)+\rho \left({W}_{b}-{W}_{a}\right)+\left(h\frac{\partial \rho }{\partial t}\right)$$

The Reynolds Equation can be expressed as follows when the film thickness does not change in the x-direction:,

5 $$\therefore \frac{\partial }{\partial x}\left(\frac{{h}^{3}}{12\mu }\frac{\partial p}{\partial x}\right)+\frac{\partial }{\partial y}\left(\frac{{h}^{3}}{12\mu }\frac{\partial p}{\partial y}\right)=\frac{\partial h}{\partial t}$$

**Fig. 3:**
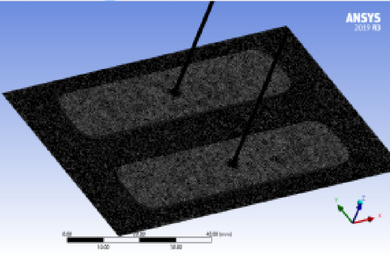
Rectangular recess in hydrostatic pad.

The FE meshed model of the fluid domain under investigation in a rectangular recess is shown in Fig. 3.

#### Numerical setup

Numerical simulations were generated using the commercial CFD software Ansys Fluent 19 R3. The analysis was implemented under steady-state, isothermal, and incompressible flow conditions. Energy, multiphase, and cavitation models were not considered, as the model configuration illustrates. A viscous laminar flow model was selected for the simulation.

- The Meshing Approach

The fluid domain was divided using the Ansys Meshing tool. Based on a convergence analysis, a uniform element size of 0.5 mm was selected for the model. The generated mesh contains 404,952 elements and 100,794 nodes.

- Convergence Criteria and Solver Settings

A pressure-based solver, which performs well for incompressible flow, was used for the simulation. The SIMPLE algorithm was selected for the pressure-velocity coupling. When the scaled residuals dropped below a predetermined level, the solution converged. The absolute convergence conditions for the continuity were set at 1e^-07^.

- Boundary Conditions:

Water is used for the internal flow of the hydrostatic bearing and is regarded as an incompressible fluid. The following boundary conditions were applied to the defined zones of the model: Pressure at the Inlet is a gauge pressure of 1,000,000 Pa (1 MPa). Outlet: With the gauge pressure set to 0 Pa, to simulate discharge to atmospheric pressure. Walls: All walls, including pocket and stationary walls, were considered fixed and immovable for this investigation, and a no-slip condition was put in place.

### Validation model

**Fig. 4:**
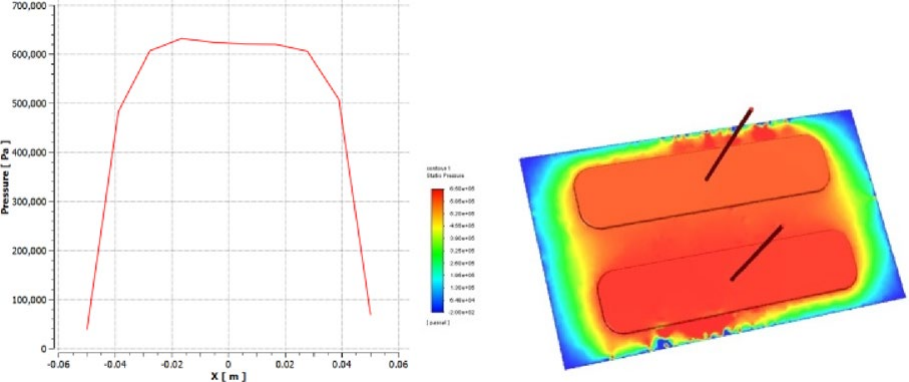
**a** Static Pressure of Recess and length, **b** Pressure distribution.

**Fig. 5:**
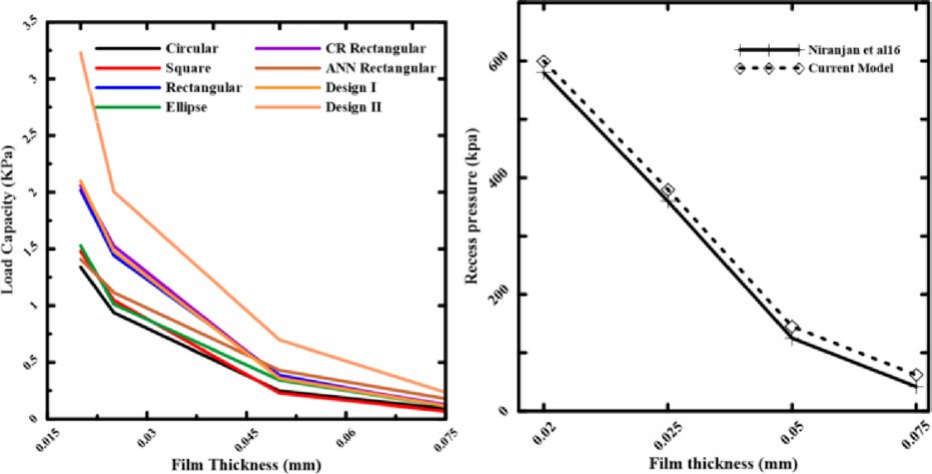
Comparison of recess pressure between the present study and the published data from [Niranjan et al^22^].

To prove the accuracy and validity of the numerical model presented in this study, a comprehensive validation was performed against the published data of [Niranjan et al^22^]. At different working film thicknesses, the comparison was done to ensure the model is reliable. Both a visual and a quantitative examination were carried out to demonstrate the agreement between the published data and the current research. Figures 4 and 5 display the numerical findings for static pressure and load capacity of the recess of the rectangular shape as reported by Niranjan et al^22^. Using ANSYS FLUENT 19.3 to validate the model. The findings indicate a respectable discrepancy between the published results and the present CFD work.

A visual comparison of the recess pressure vs film thickness is shown in Fig. 4. The current study’s results are plotted against the distinct data points from published results, as indicated by the red solid line. As can be seen, with an error of about 3%, our model’s estimates closely match the published numerical results. For a detailed quantitative assessment, the specific values at each tested condition are summarized in Table 2, showing the differences between the outcomes of our model and the published data. As can be seen, the recess pressure percentage error is a remarkably low 3.45%, indicating a nearly perfect connection and validating the accuracy of the simulation approach employed in this study.

**Table 2:** Quantitative validation of recess pressure against published data from [Niranjan et al^22^].

Film Thickness, mm Parameter	Published Result [Niranjan et al^22^] Recess Pressure, kpa	Present Study Result Recess Pressure, kpa	Percentage Error (%)
0.02	580	600	3
0.025	360	380	5
0.05	125	145	16
0.075	42	62	42

Table 2, which summarizes the validation results, demonstrates that the CFD model correctly predicts the bearing’s performance, particularly in the nominal and high-load operating regimes. With errors of just 3.45% and 5.56%, respectively, the model demonstrated exceptional accuracy for pressures of 580 kPa and 360 kPa. In the lowest film conditions, the relative percentage error increases considerably (16.0% and 47.6%). Often, a constant absolute error (usually around 20 N) is mathematically amplified when normalized by a modest reference value. Moreover, secondary physical phenomena that are not taken into consideration in the model may exert a more noticeable influence in such low-energy regimes. The model’s validity for the study’s intended scope—which focuses on performance optimization under significant operational loads—is validated by its proven good accuracy in the upper range of situations. Overall, the average error for all sites under examination was 18.16%.

### Physical model

#### Load carrying capacity

The pressure inside the bearing determines a pad’s load capacity. The bearing pad’s load-bearing capacity was calculated as follows: [23,24]:

6 $$\text{w}={\text{a}}_{\text{b}}{\text{A}}_{\text{p}} {\text{P}}_{\text{r}}$$

It is possible to determine the pad coefficient if the pressure drop across a rectangular pad’s sill is linear^21^.

7 $${a}_{b}=1-\frac{l}{L}-\frac{b}{B}=\frac{1}{2} [1+\frac{{A}_{r}}{{A}_{S}}]$$

Since Ar is the recess cross-section of bearing area, and the sill area is the same.

#### Pumping power losses

The pumping power losses will be illustrated for rectangular and circular shapes as follows:

${H}_{p}={p}_{r}q={H}_{b}{(\frac{{w}_{z}}{{A}_{p}})}^{2}\frac{{{h}_{o}}^{3}}{\mu },$ Hp = Pumping Power, q = flow rate [25]

## Numerical results

**Fig. 6:**
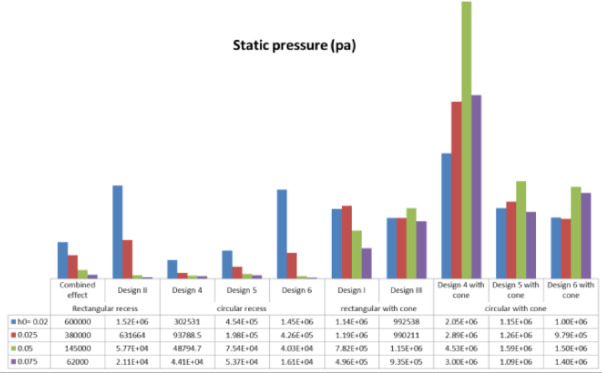
Static pressure at different film thicknesses.

The effects of pressure supply and recess shapes on the pressure distribution of a hydrostatic bearing with a rectangular pad in a steady state were investigated using 3D Navier–Stokes equations, a pressure-based solver, and a laminar model. Figure 6 displays the static pressure for each of the suggested designs using the ANSYS-CFD-19 R3 software application’s finite volume approach.

### Effect of recess conical shapes on power losses

A key factor that influences both bearing power and load-carrying capacity is film thickness. To investigate how conical recess shapes affect bearing power losses, four recesses of rectangular and circular pads, named Design II and Design 6, with film thicknesses of 0.075, 0.05, 0.025, and 0.02 mm, were chosen. Four rectangular recesses (Design II) and four conical-shaped recesses (Design III) will be used to demonstrate the load-carrying capacity. They are referred to as Design 6 & Design 6 with cone to indicate the conical influence on power losses.

**Fig. 7:**
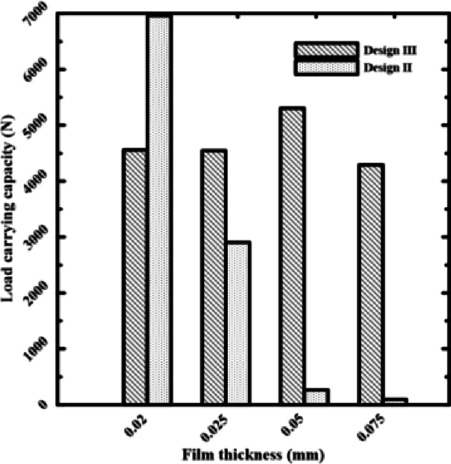
Load capacity of rectangular recess.

**Fig. 8:**
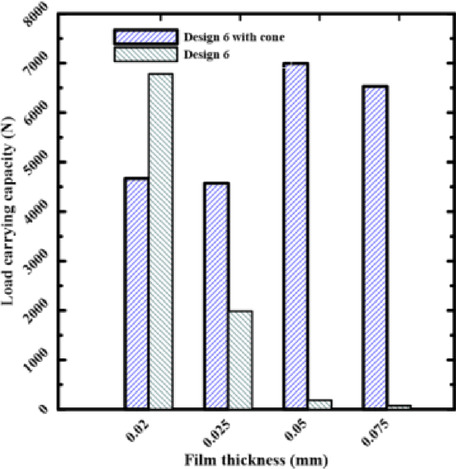
Load capacity of circular recess.

All of the examined designs show the same basic trend: as the film thickness decreases, the load-carrying capacity rises. This trend occurred due to the laws of hydraulic resistance control. The flow resistance over the bearing land is significantly increased by a thinner coating, which shortens the escape path of the compressed fluid. Because of this resistance, pressure rises over the bearing surface, increasing the net lifting force. The relationship between film thickness and load-carrying capacity is illustrated in Figs. 7 and 8. The load-carrying capacity increased as the film thickness reduced for the rectangular recess shape in Design II, which includes four recesses. The capacity value of Design III, which contains four conical-shaped recesses, ranged about 5000 N, but generally increased as the film thickness decreased. At moderate to high film thicknesses, the designs with a conical profile in Figs. 5 and 7 consistently show a higher load-carrying capability than their conventional equivalents. This illustrates how crucial the conical profile is for encouraging pressure rise even before the film thickness reaches a critical point. These findings clearly demonstrate that even when reducing film thickness is the primary strategy for increasing load capacity, geometric changes—most notably, the use of a conical shape—provide a notable performance improvement. This finding has significant applications, as it offers a straightforward method for enhancing bearing performance, even in situations where employing thin layers would not be practicable or desirable. The bearing capacity increased as the film thickness dropped for the circular recess shape in Design 6, which has four recesses. The capacity value of Design 6, which has four conical-shaped recesses, increased as the film thickness declined at high film thickness and was almost equal at low film thickness. In the two cases of rectangular and circular recesses, the load-carrying capacity rose as the film thickness dropped at conical shapes.

**Fig. 9:**
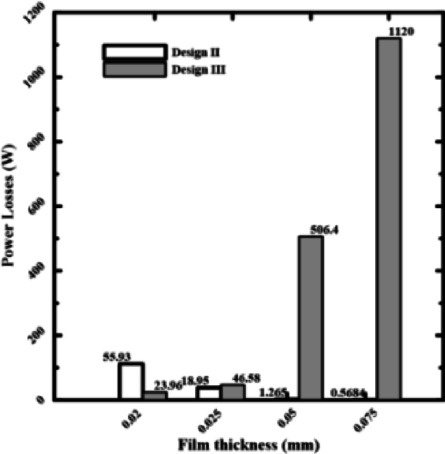
Power losses of rectangular recess for conical and non-conical shapes.

**Fig. 10:**
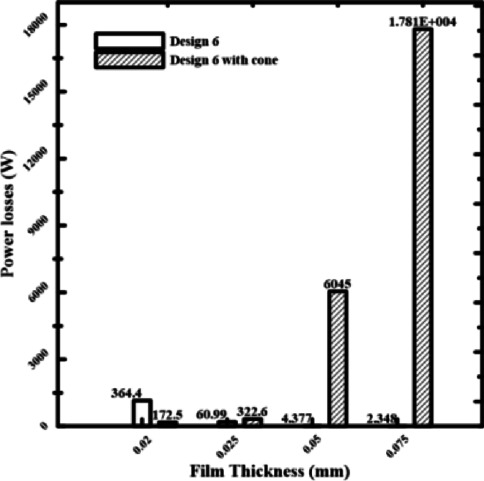
Power losses of circular recess for conical and non-conical shapes.

According to Figs. 9 and 10, bearing power losses increase when film thickness reduces in rectangular recesses without conical shapes, which is also the outcome in Yang et al^14^. However, they reduce in conical rectangular recesses. The same is true for circular recess shapes; in the case of conical recess shapes, the bearing power losses decrease with film thickness. As a consequence, the load-carrying capacity and bearing power losses both increased as the film thickness dropped. The conflict indicated above can be addressed to improve the overall bearing performance by altering the recess shape effect on power losses at minimal film thickness. As the film thickness decreased for conventional designs, both bearing power losses and load-carrying capacity increased. The findings provided here demonstrate that this performance conflict can be resolved. The special geometry of the conical recess offers the solution. Particularly, the conical profile operates as a "pre-pressurized" region, allowing the bearing to generate a high load capacity without primarily concentrating on the severe pressure drops caused by an ultra-thin layer. This results in a significant reduction of the fluid shear rate over the bearing land, which is the primary cause of viscous power loss. Data collected show that the power losses of the conical designs are an order of magnitude lower than those of traditional designs.

### Effect of the number of recesses on power losses

**Fig. 11:**
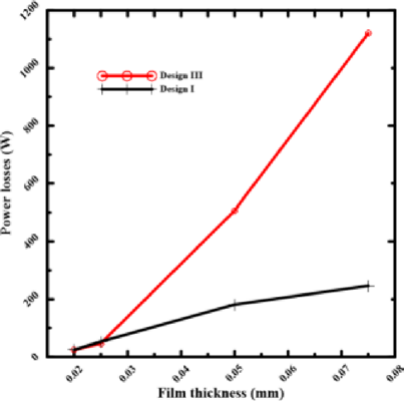
Power losses of rectangular recess for number of recess.

**Fig. 12:**
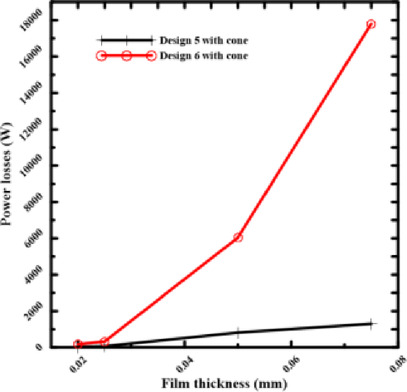
Power losses of circular recess for number of recess.

The selection of the recess number is crucial if bearing performance is to continue to improve. The pumping power losses will be shown for rectangular and circular shapes, as shown in Figs. 11 and 12, by choosing two and four recesses of conical shapes.

Pumping power losses decreased at overall film thicknesses used for this investigation in the rectangular and circular shapes when the number of recesses was reduced from four to two recesses, while maintaining the same configuration and supply pressure. At large film thicknesses, this impact is evident, but at minimal thicknesses, it is not as noticeable as at greater ones. At high film thicknesses, there is no flow resistance and a significant clearance. The main reason for power loss in this regime is the pumping power required to transport the fluid to the recesses. Reducing the number of recesses from four to two results in a smaller overall perimeter of the recesses, which lowers the total flow rate required to sustain the pressure. Because it directly causes a significant drop in pumping power, the effect is especially “evident” in this region. At minimal film thicknesses, the clearance becomes very narrow. In this case, the viscous shear power becomes the primary source of power loss due to the shearing of the fluid layer between the rotating and stationary surfaces. While reducing the number of recesses still lowers the pumping power, this reduction becomes a smaller fraction of the total power loss, which is now dominated by the massive increase in viscous shear. The effect is "not as noticeable" under this regime because of this. For systems that often operate at larger clearances or have variable operating conditions, reducing the number of recesses is a straightforward and effective technique to increase energy efficiency. The benefits of this approach remain applicable to systems designed to function effectively in the ultra-thin film regime. Still, they are overcome by the effects of optimizing the recess profile. This highlights the importance of applying an exhaustive design approach that considers the bearing’s desired operating regime.

**Fig. 13:**
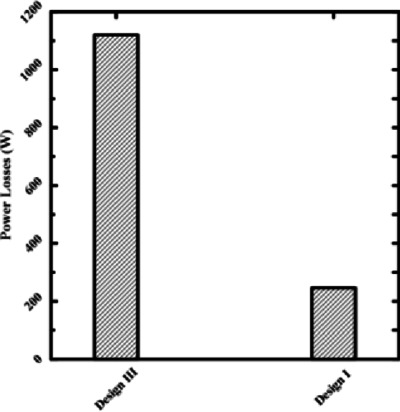
Power losses of 2 and 4 rectangular recesses at the same thickness.

**Fig. 14:**
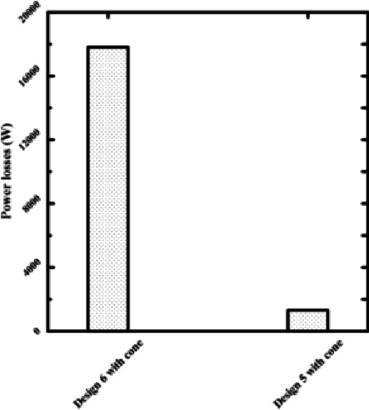
Power losses of 2 and 4 circular recesses at the same thickness.

Figures 13 and 14 make it evident that power losses will drop at 0.075 mm film thicknesses if two recesses (Design I) are chosen for rectangular bearings. The circular recess at Design 5 with the cone has the same outcome. This direct comparison demonstrates that reducing the number of recesses is one of the best ways to design hydrostatic bearings that consume less energy. For applications that don’t require an extremely high degree of stiffness in all directions, reducing the number of supply recesses is a simple and effective technique for significantly reducing operational energy costs. Resolving performance conflicts at thin film thicknesses requires consideration of other parameters, such as recess profile.

### Effect of rectangular and circular performance on power losses

The performance of the circular and rectangular shapes should be accurately evaluated for overall improvement at the same film thickness and number of conical shape recesses.

**Fig. 15:**
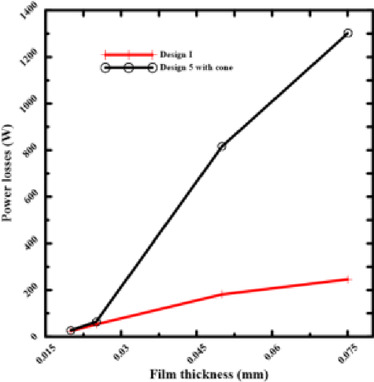
Power losses of circular and rectangular recesses at various thicknesses.

According to the illustration, in Fig. 15, bearing power losses are reduced in rectangular and circular shapes. When the film thickness and number of recesses are the same, the power loss values for rectangular shapes are lower than those for circular ones. By choosing a rectangular recess shape, bearing performance is improved and losses are reduced. The rectangular Design I performed better in this comparison because it had a better balance between the recess space and the surrounding land area. A well-designed rectangular recess can result in a more uniform and efficient pressure distribution by lowering the high flow rates that cause significant pumping power losses, which appear to predominate in the circular Design 5 with cone.

## Conclusion

This study demonstrates that the geometric design of hydrostatic thrust bearings, namely the shape and profile of the recesses, is a crucial component in enhancing their performance for high-precision applications. One of the key conclusions of the study is the finding of the fundamental conflict between load-carrying capacity and power loss. The findings show that implementing a conical recess profile effectively reduces the trade-off between higher power consumption and increased load capacity, which is typically associated with lowering film thickness. At minimum film thickness, bearings with conical recesses showed a significant reduction in power loss while maintaining a good load-carrying capacity. This method also provides new insights into the role of recess shape and number. The findings show that circular recesses generally provide lower power losses than rectangular ones under similar conditions. A more efficient method of increasing energy efficiency is to reduce the number of recesses (for instance, from four to two), as this significantly improves pumping efficiency, especially at greater film thicknesses. In conclusion, the primary contribution of this study is the development of an improved design structure for energy-efficient hydrostatic bearings: a conical recess profile is the most effective method of minimizing power loss in ultra-thin film applications, which is followed by a planned reduction in the number of recesses. By providing a good foundation for developing future-oriented hydrostatic bearings that achieve superior performance and increased energy economy, our findings solve a significant problem in the field. Even though the results clearly demonstrate the performance benefits of a conical recess profile, designers still need to consider the practical consequences of implementing this design. Conical shapes are fundamentally more difficult and costly to make than standard flat recesses, and they may require particular CNC machining techniques. As a result, there is a solution: For critical, high-precision applications (such as scientific instruments or machine tools), the significant performance gains (such as enhanced stability and decreased power loss) would likely offset the increased production costs. For low-cost, general-purpose applications, a simpler, traditional design could still be the more economical choice even when it performs poorly. This study provides designers with the quantitative data they require to balance performance requirements for their specific application with budgetary constraints. For designers, this study is significant since it creates opportunities for further research.

## Data Availability

The datasets used are available from the corresponding author on reasonable request.

## List of symbols

A: Rectangular pad area
A_e_: Effective pad area
L: Rectangular pad length
B: Rectangular pad width
b: Bearing sill width
Hp: Pumping power
P_r_: Recess pressure
Q: Flow rare
W: Load carrying capacity
a_b_: Load coefficient of bearing pad
A_p_: Pad projected area
q_b_: Flow coefficient of bearing pad
l: Bearing sill length
